# UPS: Opportunities and challenges for gastric cancer treatment

**DOI:** 10.3389/fonc.2023.1140452

**Published:** 2023-04-03

**Authors:** Hang Yang, Huihan Ai, Jialin Zhang, Jie Ma, Kangdong Liu, Zhi Li

**Affiliations:** ^1^ The Affiliated Cancer Hospital of Zhengzhou University & Henan Cancer Hospital, Zhengzhou, China; ^2^ Department of Pathophysiology, School of Basic Medical Sciences, Zhengzhou University, Zhengzhou, Henan, China; ^3^ China-US Hormel (Henan) Cancer Institute, Zhengzhou, Henan, China; ^4^ Research Center of Basic Medicine, Academy of Medical Sciences, Zhengzhou University, Zhengzhou, Henan, China

**Keywords:** UPS modulator, PROTAC, E3 ligase, gastric cancer, therapy

## Abstract

Gastric cancer remains the fourth most frequently diagnosed malignancy and the fifth leading cause of cancer-related mortality worldwide owning to the lack of efficient drugs and targets for therapy. Accumulating evidence indicates that UPS, which consists of E1, E2, and E3 enzymes and proteasome, plays an important role in the GC tumorigenesis. The imbalance of UPS impairs the protein homeostasis network during development of GC. Therefore, modulating these enzymes and proteasome may be a promising strategy for GC target therapy. Besides, PROTAC, a strategy using UPS to degrade the target protein, is an emerging tool for drug development. Thus far, more and more PROTAC drugs enter clinical trials for cancer therapy. Here, we will analyze the abnormal expression enzymes in UPS and summarize the E3 enzymes which can be developed in PROTAC so that it can contribute to the development of UPS modulator and PROTAC technology for GC therapy.

## 1 Introduction

According to the global cancer statistics, one million new gastric cancer (GC) cases and about 769,000 deaths were estimated in 2020, ranking fourth for mortality and fifth for incidence among all types (1). At present, the clinical treatments of GC mainly include surgical resection, chemotherapy, radiotherapy, and molecular targeted therapy (2, 3). Molecular targeted therapy has achieved major success in recent years with the understanding of molecular mechanisms in cancer. However, only scant targets are used to develop drugs in GC such as VEGFR-2, HER2, PD-1, etc. (4). Moreover, most of traditional small molecule inhibitors affect active site of the targets to inhibit its function. Due to the limit of drugs targets and related technology, the development of targeted therapy for GC is still limited. Thus, investigating more effective therapeutic targets and developing novel technologies for GC treatment are highly expected. Ubiquitin-proteasome system (UPS) is one of the main pathways for protein degradation in mammals, which regulate various cellular biological processes through changing the protein levels, such as cell signal transduction, cell cycle, transcription, DNA damage and repair, etc. (5, 6). Through the coordination between ubiquitin-activating enzyme E1, ubiquitin-conjugating enzyme E2 and ubiquitin-ligase E3 in UPS, target proteins modified by ubiquitin are transferred to the proteasome for degradation. Therefore, UPS is essential for maintaining the normal levels of intracellular proteins by removing damaged organelles and misfolded proteins (7). During GC development, it is commonly observed that dysfunction of UPS due to the abnormal changes of E1, E2, and E3 enzymes causes the imbalanced accumulation of large numbers of proteins. Fortunately, it has been validated by clinical success of many UPS modulators, emphasizing the therapeutic potential of this pathway (8). Besides, PROTAC (proteolysis-targeting chimeras) is the novel drug development technology using UPS to degrade target proteins, which plays an important role in the treatment of prostate cancer and breast cancer (9). Hence, to develop targeted drugs by using UPS has bright prospects for further GC treatment. This review will elaborate the research progresses of UPS modulators and PROTAC to provide a novel perspective for targeted therapy of GC.

## 2 UPS is a viable strategy in GC therapy

**Figure 1:**
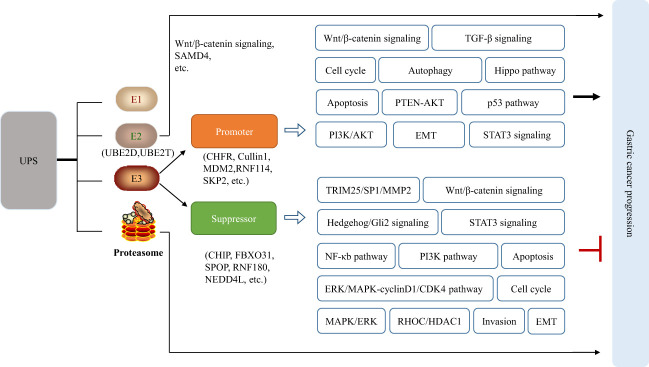
Abnormal UPS affects the normal signaling pathways in cells, leading to or inhibiting the occurrence of gastric cancer.

Compared with other cancers, such as breast cancer (HER2) and lung cancer (EGFR, ALK, ROS1), there are still no effective molecular targets for GC which lacks the dominant driver genes and epigenetics targets. Thus, researchers are turning their attention to other fields. UPS, as an important system for degradation of intracellular proteins, has attracted more attention for GC treatment. Accumulating evidence indicates that abnormal expression of E1, E2, and E3 enzymes is involved in the GC tumorigenesis, resulting in imbalance of intracellular protein homeostasis. UBE2C, UBE2T, UbcH10 are significantly upregulated in GC, which correlate with poor differentiation, high T classification, and poor prognosis (10–12). A fraction of E3 enzymes, such as MDM2, MKRN1, Cullin1, and Hakai, are overexpressed and have established oncogenic roles in gastric carcinogenesis (13–16). Numerous E3 enzymes, including FBXW7 and CHIP, have been shown to function as typical tumor suppressors in GC owing to frequent inactivating mutations or downregulated expression in GC (17). Besides, the proteasome is the primary site for protein degradation, and its activity affects UPS efficiency (18). Mechanically, abnormal UPS could lead to gastric cancer by regulating the epithelial-mesenchymal transition (EMT) through degradation of E-cadherin and N-cadherin, affecting the cell cycle through regulation of cell cycle proteins p21/p27 and cyclin D, impacting apoptosis by regulating the expression of BAX and Bcl-2 proteins. Furthermore, abnormal UPS influences the PI3K/AKT, Hippo pathway, p53 pathway, TGF-β signaling, STAT3 signaling, Wnt/β-catenin pathway, NF-κB pathway, autophagy, and so on by regulation of corresponding protein in these signaling pathway such as p53, β-catenin, SHP-1, etc. (**Figure 1**). Consequently, UPS can be considered as promising targets and it is a worth strategy by modulating the abnormality of UPS during the progression of GC for developing therapeutic drugs.

## 3 UPS modulators

**Figure 2:**
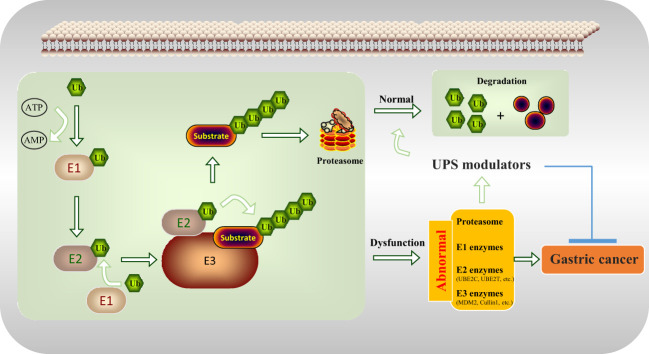
UPS modulators inhibit gastric tumorigenesis by regulating the abnormal components of the UPS including proteasome, E1 enzymes, E2 enzymes (UBE2C, UBE2T, etc.), and E3 enzymes (MDM2, Cullin1, etc.).

UPS is a complex structure involving the proteasome; and the E1, E2, and E3 enzymes (19). Derangements of UPS leads to alterations in protein homeostasis and causes many human diseases, particularly cancer (20). Here, the recent advances of UPS modulators by targeting proteasome and enzymes and how they pave the way towards GC treatment are discussed in detail below (with the working model seen in **Figure 2**).

### 3.1 Targeting proteasome for GC

The proteasome is a large multi-protein complex designed to degrade proteins specifically marked by ubiquitination (21). In 2003, the FDA approved bortezomib as the first proteasome inhibitor for treating multiple myeloma that hugely increase the survival time of patients with multiple myeloma, which has provided ample evidence that targeting the proteasome is a viable approach for the treatment of human cancer (22, 23). For GC, bortezomib suppresses proliferation *in vitro* and *in vivo*, and is more effective in GC cells with lower NF-κB activation than others (24). In addition, proteasome inhibitor MG132 can effectively reverse the multidrug resistance by promoting drug-induced apoptosis of GC cells and inhibiting the expression of p-glycoprotein, confirming the hypothesis that proteasome inhibitors may be effective chemotherapeutics for GC with multidrug resistance (25). Unfortunately, the results of the phase II clinical trial show that bortezomib as a single inhibitor is inactive in advanced or metastatic GC therapy (26, 27). Therefore, future studies of proteasome inhibitor should focus on the combination of other targeted drugs in GC therapy.

### 3.2 Targeting E1 and E2 enzymes for GC

Ubiquitin-activating enzyme E1 is the first enzyme in UPS, which mediates the activation of ubiquitin. In mammals, only two E1 enzymes including UAE and UBA6 have been discovered (28). At present, a variety of E1 enzyme inhibitors have been reported, such as PYZD-4409 and TAK-243 that inhibit the activity of UAE. PYZD-4409 not only inhibits the growth of primary acute myeloid leukemia cells *in vitro*, but also delays tumor growth in mouse models of leukemia *in vivo (* 29). In addition, TAK-243, as a new class of drugs inhibiting UAE, can induce the death of various cancer cells and attenuate the growth of xenograft models of many types of tumors (8). To date, however, there is no report on E1 enzyme inhibitors for GC due to the low specificity of inhibitors and less E1 enzymes.

Ubiquitin-conjugating enzyme E2 as the key enzyme performs the second step of the ubiquitination reaction (30). The human genome encodes around 40 different E2 enzymes (31). Accumulating evidence suggests that E2 enzymes are crucial in the occurrence and development of cancer (32). Based on the indispensability and diversity of E2 enzymes in ubiquitination, more and more inhibitors are designed for E2 than E1 enzymes. For example, the silence of UBE2D1 reduces the ubiquitination of SMAD4, inhibiting the migration of GC cells (33). Moreover, UbcH10 promotes the growth of GC cells and may represent a potential biomarker for GC (12). Besides, the novel UBE2T inhibitor controls the overactivation of Wnt/β-catenin signaling and the progression of GC by blocking RACK1 ubiquitination (34). Thus, it is attractive to develop inhibitors targeting E2 enzymes such as UBED1, UbcH10, and UBE2T for GC treatment.

### 3.3 Targeting E3 enzyme for GC

In the series of enzymatic cascades, ubiquitin-ligase enzyme E3 determines the specific recognition of target proteins and plays a key role in the functioning of the UPS (35). Compared to efforts against the E1 and E2 enzymes or proteasome, it is considered a better therapeutic target through targeting E3 enzymes for GC drug development because the E3 enzymes confer substrate specificity. Up to now, more than 600 types of E3 enzymes have been discovered in humans (36). In accordance with the roles, E3 ubiquitin ligases can be divided into two categories: tumor promoter and tumor suppressor based on their target proteins. Although many types of E3 enzymes are reported, the related research is rare in GC development. At present, about 66 kinds of E3 enzymes are involved in GC, of which 40 types exerted an oncogenic function for promoting cancer progression and 26 types play tumor-suppressive functions (**Tables 1**, **2**). Subsequently, representative E3 enzymes that play key roles in the development of GC will be summarized separately and analyzed the possibility as the drug targets.

**Table 1:** E3 enzymes as the promoter in GC.

Types	E3 enzymes	Function	Substrate	Signal	References
**RINGs**	CHFR	promoter	PARP-1	EMT, Cell cycle	(37, 38)
	Cullin1	promoter	–	Cell cycle, Apoptosis	(39)
	FBXL7	promoter	Survivin	Apoptosis	(40)
	FBXO11	promoter	–	PI3K/AKT, EMT	(41)
	FBXO2	promoter	–	EMT	(42)
	FBXO6	promoter	–	Apoptosis, Invasion	(43)
	FBXW5	promoter	–	Hippo pathway	(44)
	MDM2	promoter	p53	p53 pathway	(13)
	MKRN1	promoter	p14ARF	Senescence	(14)
	PRAJA	promoter	ELF/Smad3	TGF-β signaling	(45)
	RFWD3	promoter	p53	AKT, ERK/P38 and Slug pathways	(46)
	RNF114	promoter	–	Cell cycle	(47)
	RNF115	promoter	–	Autophagy	(48)
	RNF126	promoter	–	Cell cycle	(49)
	RNF185	promoter	JWA	Metastasis	(50)
	RNF2	promoter	–	Cell cycle	(51, 52)
	RNF31	promoter	FOXP3	Metastasis	(53)
	RNF38	promoter	SHP-1	STAT3 signaling	(54)
	RNF6	promoter	SHP-1	STAT3 signaling	(39, 55)
	SIAH1	promoter	β-catenin	Nuclear translocation of β-catenin	(56)
	SIAH2	promoter	–	Invasion	(57)
	SKP2	promoter	**-**	Cell cycle	(58)
	TRIM14	promoter	**-**	AKT signaling	(59)
	TRIM15	promoter	**-**	Invasion	(60)
	TRIM23	promoter	**-**	–	(61)
	TRIM24	promoter	**-**	Wnt/β-catenin	(62)
	TRIM29	promoter	**-**	Wnt/β-catenin	(63)
	TRIM32	promoter	–	Wnt/β-catenin, AKT	(64, 65)
	TRIM37	promoter	–	NF-κB pathway	(66)
	TRIM44	promoter	–	Metastasis	(67)
	TRIM59	promoter	p53	p53 pathway	(68)
	UBR2	promoter	–	Wnt/β-catenin	(69)
	UBR5	promoter	GKN1	–	(70, 71)
	UHRF1	promoter	–	Invasion	(72)
**HECTs**	HUWE1	promoter	TGFBR2	Invasion	(73)
	NEDD4-1	promoter	PTEN	PTEN pathway	(74)
	SMURF1	promoter	MEKK2	MEK1/2-ERK1/2	(75, 76)
	UBE3C	promoter	AXIN1	Wnt/β-catenin	(77)
	WWP1	promoter	–	PTEN-Akt	(78, 79)
	WWP2	promoter	PTEN	PTEN pathway	(80)

**Table 2:** E3 enzymes as the suppressor in GC.

Types	E3 enzymes	Function	Substrate	Signal	References
**RINGs**	CBLB	suppressor	–	Cell adhesion and Detachment	(81–83)
	CHIP	suppressor	TRAF2	NF-κB pathway	(84, 85)
	COP1	suppressor	c-Jun/p53	Invasion	(86)
	DTX1	suppressor	c-FLIP	Apoptosis	(87)
	FBX8	suppressor	–	Metastasis	(88)
	FBXL2	suppressor	FoxM1	Cell cycle	(89)
	FBXL5	suppressor	Cortactin	Invasion	(90, 91)
	FBXO21	suppressor	Nr2f2	EMT	(92)
	FBXO31	suppressor	SNAI1	EMT	(93)
	FBXW7	suppressor	MCL1/RhoA/ENO1/GFI1	Apoptosis, AKT, GSK3β	(94–97)
	MARCH8	suppressor	DR4	PI3K Pathway	(98)
	MKRN2	suppressor	PKM2	MAPK/ERK	(99)
	PRAJA2	suppressor	KSR1	MEK-ERK	(100)
	RBX1	suppressor	PRDX2	Invasion	(101)
	RNF168	suppressor	RHOC	RHOC/HDAC1	(102)
	RNF180	suppressor	RhoC	STAT3 signaling	(103–105)
	RNF181	suppressor	–	ERK/MAPK-cyclin D1/CDK4 pathway	(106)
	RNF43	suppressor	–	Wnt/β-catenin	(107, 108)
	SPOP	suppressor	–	Hedgehog/Gli2 signaling	(109)
	TRIM15	suppressor	–	Invasion	(110)
	TRIM25	suppressor	SP1	TRIM25/SP1/MMP2	(111)
	TRIM31	suppressor	–	–	(112, 113)
	ZNRF3	suppressor	–	WNT and Hedgehog signaling	(114, 115)
**HECTs**	HACE1	suppressor	–	Invasion	(116)
	ITCH	suppressor	Smad7	EMT	(117)
	NEDD4L	suppressor	–	PI3K-AKT	(118)

The growth and development of GC can be inhibited by suppressing E3 enzymes which are the tumor promoting factor. The upregulation of MDM2 and the accompanying inactivation of p53 pathway play an important role in diffuse gastric cancer (119). MiR-410 inhibits gastric cancer cells proliferation, migration, and invasion by targeting the *MDM2* gene (120). Nutlin-3, which is the MDM2 inhibitor, has anti-tumor effects in GC cells *in vitro* and *in vivo* (121). Besides, SKP2, also named FBXL1, is an F-box typed E3 ligase, which is an overexpressed and modulated malignant phenotype *via* p27 proteolysis in GC (122). Downregulation of SKP2 inhibits the growth and metastasis of GC cells (123). Moreover, UHRF1 (Ubiquitin-Like PHD And RING Finger Domain-Containing Protein 1) expression is significantly higher in GC and is an independent and significant predictor of GC prognosis (124, 125). The knockdown of UHRF1 suppresses the growth, migration, invasion, and apoptosis of GC cells *via* an ROS-associated pathway (72). To date, there is no E3 enzyme inhibitor for clinical trials in GC treatment. However, the above studies show that the development of drugs that target the promoting function of E3 enzymes is valuable for GC therapy, though researching and finding the antagonist to inhibit promoter maybe the promising therapy for GC.

Different from a tumor promoter, the expression of a tumor suppressor needs to be enhanced to inhibit the occurrence of GC. RNF43 acts as a negative regulator of the Wnt signaling pathway by mediating the ubiquitination, endocytosis, and subsequent degradation of Wnt receptor complex component Frizzled. Research shows that RNF43 that inhibits cell proliferation is significantly downregulated in the gastric carcinoma, and its expression is positively correlated with p53 and negatively correlated with Ki67 and Lgr5 protein (107). RNF43 is related to the development of GC and attenuates the stemness of GC stem-like cells through the Wnt-β/catenin signaling pathway (108). The loss of endogenous RNF43 function enhances the growth of GC (126). FBXW7 is another important E3 ligase which negatively regulates GC progression. Low levels of FBXW7 protein in primary GC contributes to malignant potential and poor prognosis (127). *In vitro* studies found that FBXW7 inhibits GC progression by inducing apoptosis and growth arrest (94). In addition, CHIP is the ubiquitin ligase which contains a tetratricopeptide repeat and a U-box, and can significantly reduce the migration and invasion of GC cells though inhibiting the NF-κB signaling pathway (84). CHIP overexpression impedes the growth of xenografts in nude mice and inhibits endothelial cell growth and tube formation (128). Based on current research, enhancing the function of E3 enzymes can inhibit the development of GC. For example, eprenetapopt, as the first targeting drug of p53 as suppressor gene, binding the cysteine residue of mutant p53 and converting to the wild-type conformation, can restore p53 function. Therefore, by restoring the function of the inactivated mutant E3 enzymes to exert tumor suppressor roles, it is an interesting research field for GC treatment.

## 4 PROTAC

**Figure 3:**
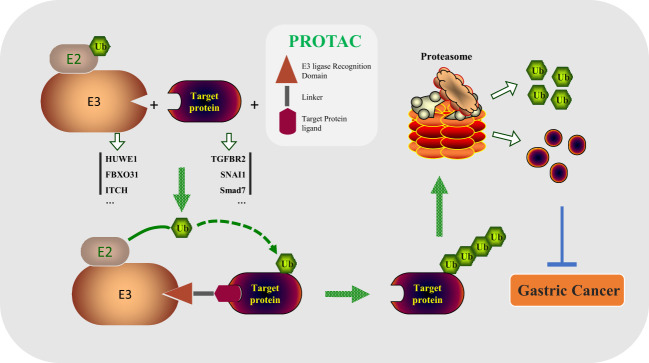
PROTAC drugs degrade important oncogenic substrates in the process of tumorigenesis by linking the E3 enzyme and substrate (HUWE1/TGFBR2, FBXO31/SNAI1, ITCH/Smad7, etc.), preventing the occurrence of gastric cancer.

Traditional small molecule drugs and antibodies play a critical role for diseases treatment by activating or inhibiting the function of the target protein, which is defined as “occupation-driven” mode (129). This mode requires higher concentration of inhibitors or monoclonal antibodies to occupy the activity site of the target so that the transduction of downstream signaling pathways is blocked (130, 131). As an emerging new technology, PROTAC is different from the “occupation-driven” mode through the UPS system to ubiquitinate and degrade the target proteins (132–134). A PROTAC includes three key parts: E3 ubiquitin ligase ligand, POI (protein of interest) ligand, and linker (135, 136). As shown in **Figure 3**, in this technology, the specific target protein is recognized by the POI ligand, and the E3 ubiquitin ligase ligand is used to recruit the specific E3 enzyme of the target protein, so that the target protein and its E3 enzyme are spatially bound together *via* a flexible chemical linker to promote the degradation of the target protein (137–139). Based on this principle, a variety of PROTAC drugs have been designed and synthesized. For example, ARV-110 is the first oral bioavailable PROTAC small molecule drug that enters clinical trials in the field of PROTAC in world, which can selectively target degradation androgen receptor (AR) to treat prostate cancer (132). In the GC process, there is an abnormal expression of protein. It is attractive by looking for E3 enzymes of these proteins to design effective PROTAC drugs for GC therapy.

### 4.1 Advantages and disadvantages of PROTAC technology

PROTAC as the novel therapeutic technology offers numerous advantages over traditional inhibition strategies (140, 141). Firstly, the dose of traditional small molecules drugs is high, which may result in toxic side effects (142). However, in PROTAC technology, by catalyzing the degradation of the target protein, lower drug concentration could achieve good degradation efficiency to overcome on-target drug toxicity (143, 144). Secondly, the UPS is the main protein degradation system and E3 ubiquitin ligases are widely expressed in a variety of cells (145). The PROTAC molecule only needs to connect the target protein and E3 enzymes together; subsequently, protein is degraded through the proteasome (146). Therefore, PROTAC technologies have broad applications for different targets. Thirdly, PROTAC degrades the protein to the basal level within a few minutes (137). However, the re-synthesis rate of most proteins is very slow so that the cell still needs the time to restore the physiological protein level; even PROTAC is completely cleared, thereby PROTAC could prolong the action time (147).

In general, the use of PROTAC drugs is associated with several disadvantages, such as worse membrane permeability and bad oral availability (148). In addition, the molecular weight of PROTAC is higher compared with traditional small molecule drugs because of its triplet form; as a result, its solubility may be poor (140). Besides, the production of PROTAC is more difficult and costly and the potential toxicity of PROTAC is longer than traditional small molecule drugs (149). Hence, the researchers need still pay attention to solve these problems for PROTAC drug development.

### 4.2 PROTAC technology in GC treatment

With GC as heterogeneous cancer, is difficult to design effective drugs using traditional targets. Patients are prone to drug resistance in the current molecular targeted drug treatment. However, PROTAC technology as a means of precise treatment could partially solve the above problems. Thus, based on the characteristics of PROTAC technology, it is a promising strategy to investigate new substrate proteins and their corresponding E3 enzyme in GC, which will provide a basis for the development of PROTAC drugs. At present, E3 enzymes are often employed for designing PROTAC including VHL, CRBN, and IAPs that belong to the RING family (9, 150, 151). Besides, cancer-related proteins such as AR, ER, BRD4, CDK4, and CDK6 are the top five studied targets for degradation (152). To date, about 10 PROTAC drugs have already entered the clinical development stages and about 110 are in pre-clinical projects worldwide. Among them, ARV-110, ARV-471, and CFT7455 that are currently the fastest clinically progressing PROTAC drugs have entered clinical phase II trials (132, 153, 154). ARV-110 is a CRBN-based PROTAC, which is designed to degrade AR for prostate cancer treatment (155). ARV-471, by degrading ER for treating breast cancer, is also a CRBN-based PROTAC (156). Moreover, CFT7455 is designed based on CRBN E3 ligases for multiple myeloma through decreasing the level of IKZF1/3 protein (157). Therefore, PROTAC technology provides the potential for development of cancer-targeted therapy drugs.

However, the development of PROTAC drugs is relatively slow in GC. According to research, ARV-825 as the PROTAC drug effectively inhibits the growth of GC cells and elevates the apoptosis through downregulation of c-MYC and PLK1, suggesting that it may be a better therapeutic strategy for GC (158), while the clinical application of ARV-825 needs to explore continually. For GC, there are more than 40 types of E3 enzymes that have been reported, but it is the focus topic how to select appropriate E3 enzymes and their targets to develop PROTAC drugs. Three main points need to be considered when choosing E3 ligase. a. Choose the E3 ligase whose main function is to induce protein degradation. b. The substrate protein of E3 enzymes should play an important role in GC development. c. The tissue and cell-level distribution of E3 enzymes should be fully considered. Here, we summarize the E3 enzymes and corresponding substrates that have been studied in GC (**Tables 1**, **2**) so as to provide the base for the development of PROTAC drugs in the future. In addition to the selection of E3 ligase and specific target protein, it is considered to select the corresponding ligand and appropriate length linker, which is the problem that restricts PROTAC technology (159). At present, most of the ligands are the thalidomide and its derivatives to recruit CRBN in clinic (157, 160). With the development of PTOTAC, a variety of E3 enzymes including AhR, clAP1, clAP2, CRBN, DCAF11, DCAF15, DCAF16, IAP, MDM2, RNF114, RNF4, VHL, and XIAP have developed corresponding ligands (147). Besides, Linker is a structure connecting the two ligands of PROTAC drugs, the length of which have an important influence on the biological activity of PROTAC drugs (137). However, there are no general rules for linker design (161). With current challenges solved, more and more E3 enzymes that are mentioned in **Tables 1** and **2** will be used to develop PROTAC drugs in the future for GC treatment. PROTAC may become another important disease treatment drug after small molecule inhibitors and monoclonal antibodies.

## 5 Conclusion

The occurrence and development of GC is often accompanied by the disorder of the UPS system, which is manifested as the abnormal expression of the E1, E2, and E3 enzymes. Targeting these abnormally expressed enzymes or proteasomes is a promising strategy for GC treatment. At present, these E3 ligases are worth considering as targets including MDM2, SKP2, UHRF1, RNF43, FBXW7, and CHIP E3 enzymes widely studied in GC. However, it is easier to inhibit the function of oncogenes than to restore the roles of tumor suppressor, so E3 enzymes as oncogenes maybe more suitable. However, there are no E3 enzyme modulators for clinical trials in GC treatment to date. Considering the extensive and complex activities regulated by ubiquitination, blocking or activating E3 ligases for GC therapy may adversely affect other normal biological process. Therefore, it remains a considerable challenge to explore and solve these problems. Moreover, PROTAC is the novel technology to develop drugs for degrading intracellular important oncogenic proteins that could accomplish precise treatment of GC through the UPS. So far, only a fraction of E3 enzymes has been shown to be suitable for PROTAC. It also needs to identify more available E3 ligases and explore the mechanisms to develop PROTAC. It is believed that with the progress of basic research and clinical trials, these problems can be solved finally. Hence, targeting the Ubiquitin-proteasome system for gastric cancer is a promising strategy to supply a gap due to lack of sufficient drug selection *via* the UPS system in GC therapy. To develop targeted drugs by using UPS has bright prospects for GC treatment in further studies.

## Author contributions

ZL and KL conceived and designed this review. HA collected the references. HY and JZ wrote the original manuscript and made the figures. ZL, JM and KL revised the manuscript. All authors contributed to the article and approved the submitted version.

## References

[1] Sung H Ferlay J Siegel RL Laversanne M Soerjomataram I Jemal A. Global cancer statistics 2020: GLOBOCAN estimates of incidence and mortality worldwide for 36 cancers in 185 countries. CA Cancer J Clin (2021) 71:209–49. doi: 10.3322/caac.21660 33538338

[2] Tan IB Ivanova T Lim KH Ong CW Deng N Lee J. Intrinsic subtypes of gastric cancer, based on gene expression pattern, predict survival and respond differently to chemotherapy. Gastroenterology (2011) 141:476–85, 485 e1-11. doi: 10.1053/j.gastro.2011.04.042 21684283 PMC3152688

[3] Barbour AP Walpole ET Mai GT Barnes EH Watson DI Ackland SP. Preoperative cisplatin, fluorouracil, and docetaxel with or without radiotherapy after poor early response to cisplatin and fluorouracil for resectable oesophageal adenocarcinoma (AGITG DOCTOR): results from a multicentre, randomised controlled phase II trial. Ann Oncol (2020) 31:236–45. doi: 10.1016/j.annonc.2019.10.019 31959340

[4] Cetin B Gumusay O Cengiz M Ozet A. Advances of molecular targeted therapy in gastric cancer. J Gastrointest Cancer (2016) 47:125–34. doi: 10.1007/s12029-016-9806-8 26875080

[5] Kerscher O Felberbaum R Hochstrasser M. Modification of proteins by ubiquitin and ubiquitin-like proteins. Annu Rev Cell Dev Biol (2006) 22:159–80. doi: 10.1146/annurev.cellbio.22.010605.093503 16753028

[6] Ulrich H D Walden H. Ubiquitin signalling in DNA replication and repair. Nat Rev Mol Cell Biol (2010) 11:479–89. doi: 10.1038/nrm2921 20551964

[7] Ding F Xiao H Wang M Xie X Hu F. The role of the ubiquitin-proteasome pathway in cancer development and treatment. Front Biosci (Landmark Ed) (2014) 19:886–95. doi: 10.2741/4254 24896323

[8] Hyer ML Milhollen MA Ciavarri J Fleming P Traore T Sappal D. A small-molecule inhibitor of the ubiquitin activating enzyme for cancer treatment. Nat Med (2018) 24:186–93. doi: 10.1038/nm.4474 29334375

[9] Li X Song Y. Proteolysis-targeting chimera (PROTAC) for targeted protein degradation and cancer therapy. J Hematol Oncol (2020) 13:50. doi: 10.1186/s13045-020-00885-3 32404196 PMC7218526

[10] Wang Y Huang F Liu M Zhao Q. UBE2C mRNA expression controlled by miR-300 and HuR determines its oncogenic role in gastric cancer. Biochem Biophys Res Commun (2021) 534:597–603. doi: 10.1016/j.bbrc.2020.11.034 33223052

[11] Yu H Xiang P Pan Q Huang Y Xie N. Ubiquitin-conjugating enzyme E2T is an independent prognostic factor and promotes gastric cancer progression. Tumour Biol (2016) 37:11723–32. doi: 10.1007/s13277-016-5020-3 27020591

[12] Yang M Qu Y Shi R Wu X Su C Hu Z. Ubiquitin-conjugating enzyme UbcH10 promotes gastric cancer growth and is a potential biomarker for gastric cancer. Oncol Rep (2016) 36:779–86. doi: 10.3892/or.2016.4906 27349176

[13] Feng C Xian Q Liu S. Micro RNA-518 inhibits gastric cancer cell growth by inducing apoptosis *via* targeting MDM2. BioMed Pharmacother (2018) 97:1595–602. doi: 10.1016/j.biopha.2017.11.091 29793321

[14] Ko A Shin JY Seo J Lee KD Lee EW Lee MS. Acceleration of gastric tumorigenesis through MKRN1-mediated posttranslational regulation of p14ARF. J Natl Cancer Inst (2012) 104:1660–72. doi: 10.1093/jnci/djs424 PMC3490844 23104211

[15] Bai J Zhou Y Chen G Zeng J Ding J Tan Y. Overexpression of Cullin1 is associated with poor prognosis of patients with gastric cancer. Hum Pathol (2011) 42:375–83. doi: 10.1016/j.humpath.2010.09.003 21190721

[16] Figueroa A Kotani H Toda Y Mazan-Mamczarz K Mueller EC Otto A. Novel roles of hakai in cell proliferation and oncogenesis. Mol Biol Cell (2009) 20:3533–42. doi: 10.1091/mbc.e08-08-0845 PMC2719571 19535458

[17] Hou Y C Deng JY. Role of E3 ubiquitin ligases in gastric cancer. World J Gastroenterol (2015) 21:786–93. doi: 10.3748/wjg.v21.i3.786 PMC4299330 25624711

[18] Goldberg AL. Development of proteasome inhibitors as research tools and cancer drugs. J Cell Biol (2012) 199:583–8. doi: 10.1083/jcb.201210077 PMC3494858 23148232

[19] Kim IS Jo WM. Effects of a proteasome inhibitor on cardiomyocytes in a pressure-overload hypertrophy rat model: An animal study. Korean J Thorac Cardiovasc Surg (2017) 50:144–52. doi: 10.5090/kjtcs.2017.50.3.144 PMC5460960 28593149

[20] Zhao Y Sun Y. Cullin-RING ligases as attractive anti-cancer targets. Curr Pharm Des (2013) 19:3215–25. doi: 10.2174/13816128113199990300 PMC4034125 23151137

[21] Gomes FP Park R Viana AG Fernandez-Costa C Topper E Kaya A. Protein signatures of seminal plasma from bulls with contrasting frozen-thawed sperm viability. Sci Rep (2020) 10:14661. doi: 10.1038/s41598-020-71015-9 32887897 PMC7474054

[22] Paramore A Frantz S. Bortezomib. Nat Rev Drug Discovery (2003) 2:611–2. doi: 10.1038/nrd1159 12908468

[23] Buac D Shen M Schmitt S Kona FR Deshmukh R Zhang Z. From bortezomib to other inhibitors of the proteasome and beyond. Curr Pharm Des (2013) 19:4025–38. doi: 10.2174/1381612811319220012 PMC3657018 23181572

[24] Nakata W Hayakawa Y Nakagawa H Sakamoto K Kinoshita H Takahashi R. Anti-tumor activity of the proteasome inhibitor bortezomib in gastric cancer. Int J Oncol (2011) 39:1529–36. doi: 10.1016/S0016-5085(11)62794-7 21785822

[25] Wang H Wang X Li Y Liao A Fu B Pan H. The proteasome inhibitor bortezomib reverses p-glycoprotein-mediated leukemia multi-drug resistance through the NF-kappaB pathway. Pharmazie (2012) 67:187–92. doi: 10.1691/ph.2012.1585 22512091

[26] Shah MA Power DG Kindler HL Holen KD Kemeny MM Ilson DH. A multicenter, phase II study of bortezomib (PS-341) in patients with unresectable or metastatic gastric and gastroesophageal junction adenocarcinoma. Invest New Drugs (2011) 29:1475–81. doi: 10.1007/s10637-010-9474-7 20574790

[27] Ocean AJ Christos P Sparano JA Shah MA Yantiss RK Cheng J. Phase II trial of bortezomib alone or in combination with irinotecan in patients with adenocarcinoma of the gastroesophageal junction or stomach. Invest New Drugs (2014) 32:542–8. doi: 10.1007/s10637-014-0070-0 PMC4047141 24526575

[28] Huang Y Xiao Y Zhang X Huang X Li Y. The emerging roles of tripartite motif proteins (TRIMs) in acute lung injury. J Immunol Res (2021) 2021:1007126. doi: 10.1155/2021/1007126 34712740 PMC8548118

[29] Xu GW Ali M Wood TE Wong D Maclean N Wang X. The ubiquitin-activating enzyme E1 as a therapeutic target for the treatment of leukemia and multiple myeloma. Blood (2010) 115:2251–9. doi: 10.1182/blood-2009-07-231191 PMC2920204 20075161

[30] Wang S Zhang G Zhang Y Song Q Chen Z Wang J. Comparative studies of mitochondrial proteomics reveal an intimate protein network of male sterility in wheat (Triticum aestivum l. ). J Exp Bot (2015) 66:6191–203. doi: 10.1093/jxb/erv322 PMC4588876 26136264

[31] Stewart MD Ritterhoff T Klevit RE Brzovic PS. E2 enzymes: more than just middle men. Cell Res (2016) 26:423–40. doi: 10.1038/cr.2016.35 PMC4822130 27002219

[32] Chen X Wang L Su X Luo SY Tang X Huang Y. Identification of potential target genes and crucial pathways in small cell lung cancer based on bioinformatic strategy and human samples. PloS One (2020) 15:e0242194. doi: 10.1371/journal.pone.0242194 33186389 PMC7665632

[33] Xie H He Y Wu Y Lu Q. Silencing of UBE2D1 inhibited cell migration in gastric cancer, decreasing ubiquitination of SMAD4. Infect Agent Cancer (2021) 16:63. doi: 10.1186/s13027-021-00402-2 34743754 PMC8574036

[34] Yu Z Jiang X Qin L Deng H Wang J Ren W. A novel UBE2T inhibitor suppresses wnt/beta-catenin signaling hyperactivation and gastric cancer progression by blocking RACK1 ubiquitination. Oncogene (2021) 40:1027–42. doi: 10.1038/s41388-020-01572-w PMC7862066 33323973

[35] Qian S Zhan X Lu M Li N Long Y Li X. Quantitative analysis of ubiquitinated proteins in human pituitary and pituitary adenoma tissues. Front Endocrinol (Lausanne) (2019) 10:328. doi: 10.3389/fendo.2019.00328 31191455 PMC6540463

[36] Zheng N Shabek N. Ubiquitin ligases: Structure, function, and regulation. Annu Rev Biochem (2017) 86:129–57. doi: 10.1146/annurev-biochem-060815-014922 28375744

[37] Yang S He F Dai M Pan J Wang J Ye B. CHFR promotes the migration of human gastric cancer cells by inducing epithelial-to-mesenchymal transition in a HDAC1-dependent manner. Onco Targets Ther (2019) 12:1075–84. doi: 10.2147/OTT.S191016 PMC6369853 30799937

[38] Kashima L Idogawa M Mita H Shitashige M Yamada T Ogi K. CHFR protein regulates mitotic checkpoint by targeting PARP-1 protein for ubiquitination and degradation. J Biol Chem (2012) 287:12975–84. doi: 10.1074/jbc.M111.321828 PMC3339944 22337872

[39] Zheng Q Zhao LY Kong Y Nan KJ Yao Y Liao ZJ. CDK-associated cullin 1 can promote cell proliferation and inhibit cisplatin-induced apoptosis in the AGS gastric cancer cell line. World J Surg Oncol (2013) 11:5. doi: 10.1186/1477-7819-11-5 23311997 PMC3585504

[40] Kamran M Long ZJ Xu D Lv SS Liu B Wang CL. Aurora kinase a regulates survivin stability through targeting FBXL7 in gastric cancer drug resistance and prognosis. Oncogenesis (2017) 6:e298. doi: 10.1038/oncsis.2016.80 28218735 PMC5337621

[41] Sun C Tao Y Gao Y Xia Y Liu Y Wang G. F-box protein 11 promotes the growth and metastasis of gastric cancer *via* PI3K/AKT pathway-mediated EMT. BioMed Pharmacother (2018) 98:416–23. doi: 10.1016/j.biopha.2017.12.088 29278851

[42] Sun X Wang T Guan ZR Zhang C Chen Y Jin J. FBXO2, a novel marker for metastasis in human gastric cancer. Biochem Biophys Res Commun (2018) 495:2158–64. doi: 10.1016/j.bbrc.2017.12.097 29269301

[43] Zhang L Hou Y Wang M Wu B Li N. A study on the functions of ubiquitin metabolic system related gene FBG2 in gastric cancer cell line. J Exp Clin Cancer Res (2009) 28:78. doi: 10.1186/1756-9966-28-78 19515249 PMC2709112

[44] Yao Y Liu Z Huang S Huang C Cao Y Li L. The E3 ubiquitin ligase, FBXW5, promotes the migration and invasion of gastric cancer through the dysregulation of the hippo pathway. Cell Death Discovery (2022) 8:79. doi: 10.1038/s41420-022-00868-y 35210431 PMC8873275

[45] Mishra L Katuri V Evans S. The role of PRAJA and ELF in TGF-beta signaling and gastric cancer. Cancer Biol Ther (2005) 4:694–9. doi: 10.4161/cbt.4.7.2015 16096365

[46] Jia J Yang Y Yan T Chen T Lu G. Down-regulation of RFWD3 inhibits cancer cells proliferation and migration in gastric carcinoma. Gen Physiol Biophys (2020) 39:363–71. doi: 10.4149/gpb_2020009 32902405

[47] Feng Z Li L Zeng Q Zhang Y Tu Y Chen W. RNF114 silencing inhibits the proliferation and metastasis of gastric cancer. J Cancer (2022) 13:565–78. doi: 10.7150/jca.62033 PMC8771524 35069903

[48] Li R Gu Z Zhang X Yu J Feng J Lou Y. RNF115 deletion inhibits autophagosome maturation and growth of gastric cancer. Cell Death Dis (2020) 11:810. doi: 10.1038/s41419-020-03011-w 32980859 PMC7519909

[49] Migita K Matsumoto S Wakatsuki K Kunishige T Nakade H Miyao S. RNF126 as a marker of prognosis and proliferation of gastric cancer. Anticancer Res (2020) 40:1367–74. doi: 10.21873/anticanres.14078 32132033

[50] Qiu D Wang Q Wang Z Chen J Yan D Zhou Y. RNF185 modulates JWA ubiquitination and promotes gastric cancer metastasis. Biochim Biophys Acta Mol Basis Dis (2018) 1864:1552–61. doi: 10.1016/j.bbadis.2018.02.013 29481911

[51] Zhang J Sun Z Han Y Yao R Yue L Xu Y. Rnf2 knockdown reduces cell viability and promotes cell cycle arrest in gastric cancer cells. Oncol Lett (2017) 13:3817–22. doi: 10.3892/ol.2017.5868 PMC5431740 28529595

[52] Yue F Peng K Zhang L Zhang J. Circ_0004104 accelerates the progression of gastric cancer by regulating the miR-539-3p/RNF2 axis. Dig Dis Sci (2021) 66:4290–301. doi: 10.1007/s10620-020-06802-5 33449226

[53] Zhu F Yi G Liu X Zhu F Zhao A Wang A. Ring finger protein 31-mediated atypical ubiquitination stabilizes forkhead box P3 and thereby stimulates regulatory T-cell function. J Biol Chem (2018) 293:20099–111. doi: 10.1074/jbc.RA118.005802 PMC6311505 30389786

[54] Zhang J Wu H Yi B Zhou J Wei L Chen Y. RING finger protein 38 induces gastric cancer cell growth by decreasing the stability of the protein tyrosine phosphatase SHP-1. FEBS Lett (2018) 592:3092–100. doi: 10.1002/1873-3468.13225 30112836

[55] Huang Z Cai Y Yang C Chen Z Sun H Xu Y. Knockdown of RNF6 inhibits gastric cancer cell growth by suppressing STAT3 signaling. Onco Targets Ther (2018) 11:6579–87. doi: 10.2147/OTT.S174846 PMC6178940 30323630

[56] Das L Kokate SB Dixit P Rath S Rout N Singh SP. Membrane-bound beta-catenin degradation is enhanced by ETS2-mediated Siah1 induction in helicobacter pylori-infected gastric cancer cells. Oncogenesis (2017) 6:e327. doi: 10.1038/oncsis.2017.26 28481365 PMC5523059

[57] Kokate SB Dixit P Poirah I Roy AD Chakraborty D Rout N. Testin and filamin-c downregulation by acetylated Siah2 increases invasiveness of helicobacter pylori-infected gastric cancer cells. Int J Biochem Cell Biol (2018) 103:14–24. doi: 10.1016/j.biocel.2018.07.012 30063986

[58] Wen Y Wang K Yang K. Inhibiting the role of Skp2 suppresses cell proliferation and tumorigenesis of human gastric cancer cells *via* the upregulation of p27kip1. Mol Med Rep (2016) 14:3917–24. doi: 10.3892/mmr.2016.5676 27572672

[59] Wang F Ruan L Yang J Zhao Q Wei W. TRIM14 promotes the migration and invasion of gastric cancer by regulating epithelialtomesenchymal transition *via* activation of AKT signaling regulated by miR1955p. Oncol Rep (2018) 40:3273–84. doi: 10.3892/or.2018.6750 PMC6196628 30272351

[60] Zhou W Chen H Ruan Y Zeng X Liu F. High expression of TRIM15 is associated with tumor invasion and predicts poor prognosis in patients with gastric cancer. J Invest Surg (2021) 34:853–61. doi: 10.1080/08941939.2019.1705443 31906745

[61] Yao Y Liu Z Guo H Huang S Zhong M Deng J. Elevated TRIM23 expression predicts poor prognosis in Chinese gastric cancer. Pathol Res Pract (2018) 214:2062–8. doi: 10.1016/j.prp.2018.10.010 30477642

[62] Fang Z Deng J Zhang L Xiang X Yu F Chen J. TRIM24 promotes the aggression of gastric cancer *via* the wnt/beta-catenin signaling pathway. Oncol Lett (2017) 13:1797–806. doi: 10.3892/ol.2017.5604 PMC5403329 28454326

[63] Qiu F Xiong JP Deng J Xiang XJ. TRIM29 functions as an oncogene in gastric cancer and is regulated by miR-185. Int J Clin Exp Pathol (2015) 8:5053–61. PMC4503071 26191199

[64] Wang J Fang Y Liu T. TRIM32 promotes the growth of gastric cancer cells through enhancing AKT activity and glucose transportation. BioMed Res Int (2020) 2020:4027627. doi: 10.1155/2020/4027627 32051827 PMC6995489

[65] Wang C Xu J Fu H Zhang Y Zhang X Yang D. TRIM32 promotes cell proliferation and invasion by activating beta-catenin signalling in gastric cancer. J Cell Mol Med (2018) 22:5020–8. doi: 10.1111/jcmm.13784 PMC6156241 30079558

[66] Fu T Ji K Jin L Zhang J Wu X Ji X. ASB16-AS1 up-regulated and phosphorylated TRIM37 to activate NF-kappaB pathway and promote proliferation, stemness, and cisplatin resistance of gastric cancer. Gastric Cancer (2021) 24:45–59. doi: 10.1007/s10120-020-01096-y 32572790

[67] Kashimoto K Komatsu S Ichikawa D Arita T Konishi H Nagata H. Overexpression of TRIM44 contributes to malignant outcome in gastric carcinoma. Cancer Sci (2012) 103:2021–6. doi: 10.1111/j.1349-7006.2012.02407.x PMC7659272 22862969

[68] Zhou Z Ji Z Wang Y Li J Cao H Zhu HH. TRIM59 is up-regulated in gastric tumors, promoting ubiquitination and degradation of p53. Gastroenterology (2014) 147:1043–54. doi: 10.1053/j.gastro.2014.07.021 25046164

[69] Mao J Liang Z Zhang B Yang H Li X Fu H. UBR2 enriched in p53 deficient mouse bone marrow mesenchymal stem cell-exosome promoted gastric cancer progression *via* wnt/beta-catenin pathway. Stem Cells (2017) 35:2267–79. doi: 10.1002/stem.2702 28895255

[70] Yang M Jiang N Cao QW Ma MQ Sun Q. The E3 ligase UBR5 regulates gastric cancer cell growth by destabilizing the tumor suppressor GKN1. Biochem Biophys Res Commun (2016) 478:1624–9. doi: 10.1016/j.bbrc.2016.08.170 27590582

[71] Ding F Zhu X Song X Yuan P Ren L Chai C. UBR5 oncogene as an indicator of poor prognosis in gastric cancer. Exp Ther Med (2020) 20:7. doi: 10.3892/etm.2020.9135 32934672 PMC7471948

[72] Zhang H Song Y Yang C Wu X. UHRF1 mediates cell migration and invasion of gastric cancer. Biosci Rep (2019) 26(Suppl1):113–819. doi: 10.1042/BSR20181065 PMC6435548 30352833

[73] He Y Zhou J Wan Q. The E3 ligase HUWE1 mediates TGFBR2 ubiquitination and promotes gastric cancer cell proliferation, migration, and invasion. Invest New Drugs (2021) 39:713–23. doi: 10.1007/s10637-020-01041-x 33405091

[74] Kim SS Yoo NJ Jeong EG Kim MS Lee SH. Expression of NEDD4-1, a PTEN regulator, in gastric and colorectal carcinomas. APMIS (2008) 116:779–84. doi: 10.1111/j.1600-0463.2008.00999.x 19024597

[75] Ji CD Wang YX Xiang DF Liu Q Zhou ZH Qian F. Kir2.1 interaction with Stk38 promotes invasion and metastasis of human gastric cancer by enhancing MEKK2-MEK1/2-ERK1/2 signaling. Cancer Res (2018) 78:3041–53. doi: 10.1158/0008-5472.CAN-17-3776 PMC8111788 29549164

[76] Tao Y Sun C Zhang T Song Y. SMURF1 promotes the proliferation, migration and invasion of gastric cancer cells. Oncol Rep (2017) 38:1806–14. doi: 10.3892/or.2017.5825 28731194

[77] Zhang Y Xu J Fu H Wei Z Yang D Yan R. UBE3C promotes proliferation and inhibits apoptosis by activating the beta-catenin signaling *via* degradation of AXIN1 in gastric cancer. Carcinogenesis (2021) 42:285–93. doi: 10.1093/carcin/bgaa098 32930707

[78] Li Q Li Z Wei S Wang W Chen Z Zhang L. Overexpression of miR-584-5p inhibits proliferation and induces apoptosis by targeting WW domain-containing E3 ubiquitin protein ligase 1 in gastric cancer. J Exp Clin Cancer Res (2017) 36:59. doi: 10.1186/s13046-017-0532-2 28431583 PMC5401563

[79] Zhang L Wu Z Ma Z Liu H Wu Y Zhang Q. WWP1 as a potential tumor oncogene regulates PTEN-akt signaling pathway in human gastric carcinoma. Tumour Biol (2015) 36:787–98. doi: 10.1007/s13277-014-2696-0 25293520

[80] Wang K Liu J Zhao X Li H Luo G Yu Y. WWP2 regulates proliferation of gastric cancer cells in a PTEN-dependent manner. Biochem Biophys Res Commun (2020) 521:652–9. doi: 10.1016/j.bbrc.2019.10.179 31677789

[81] Fan Y Qu X Ma Y Qu J Liu Y Hu X. Cbl-b accelerates trypsin-induced cell detachment through ubiquitination and degradation of proline-rich tyrosine kinase 2. Tumour Biol (2014) 35:11129–35. doi: 10.1007/s13277-014-2296-z 25099615

[82] Che X Zhang Y Qu X Guo T Ma Y Li C. The E3 ubiquitin ligase cbl-b inhibits tumor growth in multidrug-resistant gastric and breast cancer cells. Neoplasma (2017) 64:887–92. doi: 10.4149/neo_2017_610 28895413

[83] Fan Y Che X Hou K Zhang M Wen T Qu X. MiR-940 promotes the proliferation and migration of gastric cancer cells through up-regulation of programmed death ligand-1 expression. Exp Cell Res (2018) 373:180–7. doi: 10.1016/j.yexcr.2018.10.011 30367831

[84] Liu F Zhou J Zhou P Chen W Guo F. The ubiquitin ligase CHIP inactivates NF-kappaB signaling and impairs the ability of migration and invasion in gastric cancer cells. Int J Oncol (2015) 46:2096–106. doi: 10.3892/ijo.2015.2893 25672477

[85] Dai H Chen H Xu J Zhou J Shan Z Yang H. The ubiquitin ligase CHIP modulates cellular behaviors of gastric cancer cells by regulating TRAF2. Cancer Cell Int (2019) 19:132. doi: 10.1186/s12935-019-0832-z 31130821 PMC6524225

[86] Sawada G Ueo H Matsumura T Uchi R Ishibashi M Mima K. Loss of COP1 expression determines poor prognosisin patients with gastric cancer. Oncol Rep (2013) 30:1971–5. doi: 10.3892/or.2013.2664 23933908

[87] Hsu TS Mo ST Hsu PN Lai MZ. C-FLIP is a target of the E3 ligase deltex1 in gastric cancer. Cell Death Dis (2018) 9:135. doi: 10.1038/s41419-017-0165-6 29374180 PMC5833402

[88] Wu P Wang F Wang Y Men H Zhu X He G. Significance of FBX8 in progression of gastric cancer. Exp Mol Pathol (2015) 98:360–6. doi: 10.1016/j.yexmp.2015.03.015 25801334

[89] Li LQ Pan D Chen H Zhang L Xie WJ. F-box protein FBXL2 inhibits gastric cancer proliferation by ubiquitin-mediated degradation of forkhead box M1. FEBS Lett (2016) 590:445–52. doi: 10.1002/1873-3468.12071 26790640

[90] Wu W Ding H Cao J Zhang W. FBXL5 inhibits metastasis of gastric cancer through suppressing Snail1. Cell Physiol Biochem (2015) 35:1764–72. doi: 10.1159/000373988 25832584

[91] Cen G Ding HH Liu B Wu WD. FBXL5 targets cortactin for ubiquitination-mediated destruction to regulate gastric cancer cell migration. Tumour Biol (2014) 35:8633–8. doi: 10.1007/s13277-014-2104-9 24867096

[92] Jiang Y Liu X Shen R Gu X Qian W. Fbxo21 regulates the epithelial-to-mesenchymal transition through ubiquitination of Nr2f2 in gastric cancer. J Cancer (2021) 12:1421–30. doi: 10.7150/jca.49674 PMC7847638 33531987

[93] Zou S Ma C Yang F Xu X Jia J Liu Z. FBXO31 suppresses gastric cancer EMT by targeting Snail1 for proteasomal degradation. Mol Cancer Res (2018) 16:286–95. doi: 10.1158/1541-7786.MCR-17-0432 29117943

[94] Li H Wang Z Zhang W Qian K Xu W Zhang S. Fbxw7 regulates tumor apoptosis, growth arrest and the epithelial-to-mesenchymal transition in part through the RhoA signaling pathway in gastric cancer. Cancer Lett (2016) 370:39–55. doi: 10.1016/j.canlet.2015.10.006 26458995

[95] Li C Deng C Pan G Wang X Zhang K Dong Z. Lycorine hydrochloride inhibits cell proliferation and induces apoptosis through promoting FBXW7-MCL1 axis in gastric cancer. J Exp Clin Cancer Res (2020) 39:230. doi: 10.1186/s13046-020-01743-3 33126914 PMC7602321

[96] Deng T Shen P Li A Zhang Z Yang H Deng X. CCDC65 as a new potential tumor suppressor induced by metformin inhibits activation of AKT1 *via* ubiquitination of ENO1 in gastric cancer. Theranostics (2021) 11:8112–28. doi: 10.7150/thno.54961 PMC8315052 34335983

[97] Kuai X Li L Chen R Wang K Chen M Cui B. SCF(FBXW7)/GSK3beta-mediated GFI1 degradation suppresses proliferation of gastric cancer cells. Cancer Res (2019) 79:4387–98. doi: 10.1158/0008-5472.CAN-18-4032 31289136

[98] Yin J Ji Z Hong Y Song Z Hu N Zhuang M. Sh-MARCH8 inhibits tumorigenesis *via* PI3K pathway in gastric cancer. Cell Physiol Biochem (2018) 49:306–21. doi: 10.1159/000492882 30138931

[99] Liu Z Xiang S Guo X Zhou J Liao L Kou J. MKRN2 inhibits the proliferation of gastric cancer by downregulating PKM2. Aging (Albany NY) (2022) 13. doi: 10.18632/aging.203643 PMC8908937 35196650

[100] Zhao Z Zhu L Xing Y Zhang Z. Praja2 suppresses the growth of gastric cancer by ubiquitylation of KSR1 and inhibiting MEK-ERK signal pathways. Aging (Albany NY) (2022) 14(4):2004–13. doi: 10.18632/aging.202356 PMC7906149 33461174

[101] Chen X Wang Y Zang W Du Y Li M Zhao G. miR-194 targets RBX1 gene to modulate proliferation and migration of gastric cancer cells. Tumour Biol (2015) 36:2393–401. doi: 10.1007/s13277-014-2849-1 25412959

[102] Xu Y Feng Y Sun Z Li Q. RNF168 promotes RHOC degradation by ubiquitination to restrain gastric cancer progression *via* decreasing HDAC1 expression. Biochem Biophys Res Commun (2021) 557:135–42. doi: 10.1016/j.bbrc.2021.03.123 33865221

[103] Xie XM Deng JY Hou YC Cui JL Wu WP Ying GG. Evaluating the clinical feasibility: The direct bisulfite genomic sequencing for examination of methylated status of E3 ubiquitin ligase RNF180 DNA promoter to predict the survival of gastric cancer. Cancer biomark (2015) 15:259–65. doi: 10.3233/CBM-150466 PMC12964677 25769451

[104] Wu Z Liu H Sun W Du Y He W Guo S. RNF180 mediates STAT3 activity by regulating the expression of RhoC *via* the proteasomal pathway in gastric cancer cells. Cell Death Dis (2020) 11:881. doi: 10.1038/s41419-020-03096-3 33082325 PMC7575565

[105] Cheung KF Lam CN Wu K Ng EK Chong WW Cheng AS. Characterization of the gene structure, functional significance, and clinical application of RNF180, a novel gene in gastric cancer. Cancer (2012) 118:947–59. doi: 10.1002/cncr.26189 21717426

[106] Wang S Wang X Gao Y Peng Y Dong N Xie Q. RN181 is a tumour suppressor in gastric cancer by regulation of the ERK/MAPK-cyclin D1/CDK4 pathway. J Pathol (2019) 248:204–16. doi: 10.1002/path.5246 PMC6593865 30714150

[107] Niu L Qin HZ Xi HQ Wei B Xia SY Chen L. RNF43 inhibits cancer cell proliferation and could be a potential prognostic factor for human gastric carcinoma. Cell Physiol Biochem (2015) 36:1835–46. doi: 10.1159/000430154 26184844

[108] Gao Y Cai A Xi H Li J Xu W Zhang Y. Ring finger protein 43 associates with gastric cancer progression and attenuates the stemness of gastric cancer stem-like cells *via* the wnt-beta/catenin signaling pathway. Stem Cell Res Ther (2017) 8:98. doi: 10.1186/s13287-017-0548-8 28446252 PMC5406878

[109] Zeng C Wang Y Lu Q Chen J Zhang J Liu T. SPOP suppresses tumorigenesis by regulating Hedgehog/Gli2 signaling pathway in gastric cancer. J Exp Clin Cancer Res (2014) 33:75. doi: 10.1186/s13046-014-0075-8 25204354 PMC4172815

[110] Chen W Lu C Hong J. TRIM15 exerts anti-tumor effects through suppressing cancer cell invasion in gastric adenocarcinoma. Med Sci Monit (2018) 24:8033–41. doi: 10.12659/MSM.911142 PMC6238583 30412518

[111] Chen JJ Ren YL Shu CJ Zhang Y Chen MJ Xu J. JP3, an antiangiogenic peptide, inhibits growth and metastasis of gastric cancer through TRIM25/SP1/MMP2 axis. J Exp Clin Cancer Res (2020) 39:118. doi: 10.1186/s13046-020-01617-8 32576271 PMC7310436

[112] Sugiura T. The cellular level of TRIM31, an RBCC protein overexpressed in gastric cancer, is regulated by multiple mechanisms including the ubiquitin-proteasome system. Cell Biol Int (2011) 35:657–61. doi: 10.1042/CBI20100772 21231912

[113] Sugiura T Miyamoto K. Characterization of TRIM31, upregulated in gastric adenocarcinoma, as a novel RBCC protein. J Cell Biochem (2008) 105:1081–91. doi: 10.1002/jcb.21908 18773414

[114] Qin H Cai A Xi H Yuan J Chen L. ZnRF3 induces apoptosis of gastric cancer cells by antagonizing wnt and hedgehog signaling. Panminerva Med (2015) 57:167–75. doi: 10.1007/s12013-015-0607-7 25923840

[115] Zhou Y Lan J Wang W Shi Q Lan Y Cheng Z. ZNRF3 acts as a tumour suppressor by the wnt signalling pathway in human gastric adenocarcinoma. J Mol Histol (2013) 44:555–63. doi: 10.1007/s10735-013-9504-9 23504200

[116] Chen YL Li DP Jiang HY Yang Y Xu LL Zhang SC. Overexpression of HACE1 in gastric cancer inhibits tumor aggressiveness by impeding cell proliferation and migration. Cancer Med (2018) 7:2472–84. doi: 10.1002/cam4.1496 PMC6010910 29673126

[117] Gen Y Yasui K Kitaichi T Iwai N Terasaki K Dohi O. ASPP2 suppresses invasion and TGF-beta1-induced epithelial-mesenchymal transition by inhibiting Smad7 degradation mediated by E3 ubiquitin ligase ITCH in gastric cancer. Cancer Lett (2017) 398:52–61. doi: 10.1016/j.canlet.2017.04.002 28400336

[118] Jiang X Zhang S Yin Z Sheng Y Yan Q Sun R. The correlation between NEDD4L and HIF-1alpha levels as a gastric cancer prognostic marker. Int J Med Sci (2019) 16:1517–24. doi: 10.7150/ijms.34646 PMC6818201 31673244

[119] Gunther T Schneider-Stock R Hackel C Kasper HU Pross M Hackelsberger A. Mdm2 gene amplification in gastric cancer correlation with expression of Mdm2 protein and p53 alterations. Mod Pathol (2000) 13:621–6. doi: 10.1038/modpathol.3880107 10874665

[120] Shen J Niu W Zhou M Zhang H Ma J Wang L. MicroRNA-410 suppresses migration and invasion by targeting MDM2 in gastric cancer. PloS One (2014) 9:e104510. doi: 10.1371/journal.pone.0104510 25136862 PMC4138091

[121] Endo S Yamato K Hirai S Moriwaki T Fukuda K Suzuki H. Potent *in vitro* and *in vivo* antitumor effects of MDM2 inhibitor nutlin-3 in gastric cancer cells. Cancer Sci (2011) 102:605–13. doi: 10.1111/j.1349-7006.2010.01821.x 21205074

[122] Masuda TA Inoue H Sonoda H Mine S Yoshikawa Y Nakayama K. Clinical and biological significance of s-phase kinase-associated protein 2 (Skp2) gene expression in gastric carcinoma: modulation of malignant phenotype by Skp2 overexpression, possibly *via* p27 proteolysis. Cancer Res (2002) 62:3819–25. 12097295

[123] Wei Z Jiang X Liu F Qiao H Zhou B Zhai B. Downregulation of Skp2 inhibits the growth and metastasis of gastric cancer cells *in vitro* and in vivo. Tumour Biol (2013) 34:181–92. doi: 10.1007/s13277-012-0527-8 23229098

[124] Zhou L Shang Y Jin Z Zhang W Lv C Zhao X. UHRF1 promotes proliferation of gastric cancer *via* mediating tumor suppressor gene hypermethylation. Cancer Biol Ther (2015) 16:1241–51. doi: 10.1080/15384047.2015.1056411 PMC4622020 26147747

[125] Ge M Gui Z Wang X Yan F. Analysis of the UHRF1 expression in serum and tissue for gastric cancer detection. Biomarkers (2015) 20:183–8. doi: 10.3109/1354750X.2015.1061599 26161699

[126] Niu L Qin HZ Xi HQ Wei B Xia SY Chen L. Loss of endogenous RNF43 function enhances proliferation and tumour growth of intestinal and gastric cells. Carcinogenesis (2019) 40:551–9. doi: 10.1093/carcin/bgy152 30380024

[127] Yokobori T Mimori K Iwatsuki M Ishii H Onoyama I Fukagawa T. p53-altered FBXW7 expression determines poor prognosis in gastric cancer cases. Cancer Res (2009) 69:3788–94. doi: 10.1158/0008-5472.CAN-08-2846 19366810

[128] Wang S Wu X Zhang J Chen Y Xu J Xia X. CHIP functions as a novel suppressor of tumour angiogenesis with prognostic significance in human gastric cancer. Gut (2013) 62:496–508. doi: 10.1136/gutjnl-2011-301522 22535373

[129] Moon S Lee BH. Chemically induced cellular proteolysis: An emerging therapeutic strategy for undruggable targets. Mol Cells (2018) 41:933–42. doi: 10.14348/molcells.2018.0372 PMC6277563 30486612

[130] Zeng S Huang W Zheng X Liyan C Zhang Z Wang J. Proteolysis targeting chimera (PROTAC) in drug discovery paradigm: Recent progress and future challenges. Eur J Med Chem (2021) 210:112981. doi: 10.1016/j.ejmech.2020.112981 33160761

[131] An S Fu L. Small-molecule PROTACs: An emerging and promising approach for the development of targeted therapy drugs. EBioMedicine (2018) 36:553–62. doi: 10.1016/j.ebiom.2018.09.005 PMC6197674 30224312

[132] Qi SM Dong J Xu ZY Cheng XD Zhang WD Qin JJ. PROTAC: An effective targeted protein degradation strategy for cancer therapy. Front Pharmacol (2021) 12:692574. doi: 10.3389/fphar.2021.692574 34025443 PMC8138175

[133] Liu Z Wang P Chen H Wold EA Tian B Brasier AR. Drug discovery targeting bromodomain-containing protein 4. J Med Chem (2017) 60:4533–58. doi: 10.1021/acs.jmedchem.6b01761 PMC5464988 28195723

[134] Zhong L Li Y Xiong L Wang W Wu M Yuan T. Small molecules in targeted cancer therapy: advances, challenges, and future perspectives. Signal Transduct Target Ther (2021) 6:201. doi: 10.1038/s41392-021-00572-w 34054126 PMC8165101

[135] Zheng M Huo J Gu X Wang Y Wu C Zhang Q. Rational design and synthesis of novel dual PROTACs for simultaneous degradation of EGFR and PARP. J Med Chem (2021) 64:7839–52. doi: 10.1021/acs.jmedchem.1c00649 34038131

[136] Yu F Cai M Shao L. Targeting protein kinases degradation by PROTACs. Front Chem (2021) 9:679120. doi: 10.3389/fchem.2021.679120 34277564 PMC8279777

[137] Wang Y Jiang X Feng F Liu W Sun H. Degradation of proteins by PROTACs and other strategies. Acta Pharm Sin B (2020) 10:207–38. doi: 10.1016/j.apsb.2019.08.001 PMC7016280 32082969

[138] Chen S Liu Y.Zhou H. Advances in the development ubiquitin-specific peptidase (USP) inhibitors. Int J Mol Sci (2021) 22(9):4546. doi: 10.3390/ijms22094546 33925279 PMC8123678

[139] Bhaduri U Merla G. Ubiquitination, biotech startups, and the future of TRIM family proteins: A TRIM-endous opportunity. Cells (2021) 10(5):1015. doi: 10.3390/cells10051015 33923045 PMC8146955

[140] Weng G Shen C Cao D Gao J Dong X He Q. PROTAC-DB: an online database of PROTACs. Nucleic Acids Res (2021) 49:D1381–7. doi: 10.1093/nar/gkaa807 PMC7778940 33010159

[141] Veggiani G Gerpe MCR Sidhu SS Zhang W. Emerging drug development technologies targeting ubiquitination for cancer therapeutics. Pharmacol Ther (2019) 199:139–54. doi: 10.1016/j.pharmthera.2019.03.003 PMC7112620 30851297

[142] Salehi B Selamoglu Z KSM M Pezzani R Redaelli M Cho WC. Liposomal cytarabine as cancer therapy: From chemistry to medicine. Biomolecules (2019) 9(12):773. doi: 10.3390/biom9120773 31771220 PMC6995526

[143] Cromm P M Crews CM. Targeted protein degradation: from chemical biology to drug discovery. Cell Chem Biol (2017) 24:1181–90. doi: 10.1016/j.chembiol.2017.05.024 PMC5610075 28648379

[144] He Y Zhang X Chang J Kim HN Zhang P Wang Y. Using proteolysis-targeting chimera technology to reduce navitoclax platelet toxicity and improve its senolytic activity. Nat Commun (2020) 11:1996. doi: 10.1038/s41467-020-15838-0 32332723 PMC7181703

[145] Fan C L Liu TB. The vacuolar morphogenesis protein Vam6-like protein Vlp1 is required for pathogenicity of cryptococcus neoformans. J Fungi (Basel) (2021) 7(6):418. doi: 10.3390/jof7060418 34072011 PMC8228526

[146] Song X Zhong H Qu X Yang L Jiang B. Two novel strategies to overcome the resistance to ALK tyrosine kinase inhibitor drugs: Macrocyclic inhibitors and proteolysis-targeting chimeras. MedComm (2020) (2021) 2:341–50. doi: 10.1002/mco2.42 PMC8554663 34766150

[147] Khan S He Y Zhang X Yuan Y Pu S Kong Q. PROteolysis TArgeting chimeras (PROTACs) as emerging anticancer therapeutics. Oncogene (2020) 39:4909–24. doi: 10.1038/s41388-020-1336-y PMC7319888 32475992

[148] Guo WH Qi X Yu X Liu Y Chung CI Bai F. Enhancing intracellular accumulation and target engagement of PROTACs with reversible covalent chemistry. Nat Commun (2020) 11:4268. doi: 10.1038/s41467-020-17997-6 32848159 PMC7450057

[149] Pettersson M Crews CM. PROteolysis TArgeting chimeras (PROTACs) - past, present and future. Drug Discovery Today Technol (2019) 31:15–27. doi: 10.1016/j.ddtec.2019.01.002 PMC6578591 31200855

[150] Yin S Liu L.Gan W. The roles of post-translational modifications on mTOR signaling. Int J Mol Sci (2021) 22(4):1784. doi: 10.3390/ijms22041784 33670113 PMC7916890

[151] Zhang Z Chang X Zhang C Zeng S Liang M Ma Z. Identification of probe-quality degraders for Poly(ADP-ribose) polymerase-1 (PARP-1). J Enzyme Inhib Med Chem (2020) 35:1606–15. doi: 10.1080/14756366.2020.1804382 PMC7470090 32779949

[152] Poirier JT George J Owonikoko TK Berns A Brambilla E Byers LA. New approaches to SCLC therapy: From the laboratory to the clinic. J Thorac Oncol (2020) 15:520–40. doi: 10.1016/j.jtho.2020.01.016 PMC7263769 32018053

[153] Wang C Zhang Y Wu Y Xing D. Developments of CRBN-based PROTACs as potential therapeutic agents. Eur J Med Chem (2021) 225:113749. doi: 10.1016/j.ejmech.2021.113749 34411892

[154] Berdeja J Ailawadhi S Horwitz SM Matous JV Mehta-Shah N Martin T. A phase 1 study of CFT7455, a novel degrader of IKZF1/3, in multiple myeloma and non-Hodgkin lymphoma. Blood (2021) 138:1675–5. doi: 10.1182/blood-2021-153575

[155] Maneiro MA Forte N Shchepinova MM Kounde CS Chudasama V Baker JR. Antibody-PROTAC conjugates enable HER2-dependent targeted protein degradation of BRD4. ACS Chem Biol (2020) 15:1306–12. doi: 10.1021/acschembio.0c00285 PMC7309268 32338867

[156] Shah VV Duncan AD Jiang S Stratton SA Allton KL Yam C. Mammary-specific expression of Trim24 establishes a mouse model of human metaplastic breast cancer. Nat Commun (2021) 12:5389. doi: 10.1038/s41467-021-25650-z 34508101 PMC8433435

[157] Cheng J Guo J North BJ Tao K Zhou P Wei W. The emerging role for cullin 4 family of E3 ligases in tumorigenesis. Biochim Biophys Acta Rev Cancer (2019) 1871:138–59. doi: 10.1016/j.bbcan.2018.11.007 PMC7179951 30602127

[158] Liao X Qian X Zhang Z Tao Y Li Z Zhang Q. ARV-825 demonstrates antitumor activity in gastric cancer *via* MYC-targets and G2M-checkpoint signaling pathways. Front Oncol (2021) 11:753119. doi: 10.3389/fonc.2021.753119 34733788 PMC8559897

[159] Lebraud H Wright DJ Johnson CN Heightman TD. Protein degradation by in-cell self-assembly of proteolysis targeting chimeras. ACS Cent Sci (2016) 2:927–34. doi: 10.1021/acscentsci.6b00280 PMC5200928 28058282

[160] Yang K Zhao Y Nie X Wu H Wang B Almodovar-Rivera CM. A cell-based target engagement assay for the identification of cereblon E3 ubiquitin ligase ligands and their application in HDAC6 degraders. Cell Chem Biol (2020) 27:866–876.e8. doi: 10.1016/j.chembiol.2020.04.008 32413286 PMC7368820

[161] Pulecio J Verma N Mejia-Ramirez E Huangfu D Raya A. CRISPR/Cas9-based engineering of the epigenome. Cell Stem Cell (2017) 21:431–47. doi: 10.1016/j.stem.2017.09.006 PMC6205890 28985525

